# Fatty Acid Amide Hydrolase (FAAH) Inhibition Plays a Key Role in Counteracting Acute Lung Injury

**DOI:** 10.3390/ijms23052781

**Published:** 2022-03-03

**Authors:** Tiziana Genovese, Andrea Duranti, Ramona D’Amico, Roberta Fusco, Daniela Impellizzeri, Alessio Filippo Peritore, Rosalia Crupi, Enrico Gugliandolo, Salvatore Cuzzocrea, Rosanna Di Paola, Rosalba Siracusa, Marika Cordaro

**Affiliations:** 1 Department of Chemical, Biological, Pharmaceutical and Environmental Sciences, University of Messina, Viale Ferdinando Stagno D’Alcontres 31, 98166 Messina, Italy; tgenovese@unime.it (T.G.); rdamico@unime.it (R.D.); dimpellizzeri@unime.it (D.I.); aperitore@unime.it (A.F.P.); rsiracusa@unime.it (R.S.); 2 Department of Biomolecular Sciences, University of Urbino, Carlo Bo Piazza del Rinascimento 6, 61029 Urbino, Italy; andrea.duranti@uniurb.it; 3 Department of Clinical and Experimental Medicine, University of Messina, Via Consolare Valeria, 98125 Messina, Italy; rfusco@unime.it; 4 Department of Veterinary Sciences, University of Messina, 98168 Messina, Italy; rcrupi@unime.it (R.C.); egugliandolo@unime.it (E.G.); 5 Department of Pharmacological and Physiological Science, Saint Louis University School of Medicine, Saint Louis, MO 63104, USA; 6 Department of Biomedical and Dental Sciences and Morphofunctional Imaging, University of Messina, Via Consolare Valeria, 98125 Messina, Italy; cordarom@unime.it

**Keywords:** acute lung injury, carrageenan, inflammation, fatty acid amide hydrolase, endocannabinoids

## Abstract

Acute lung injury (ALI) is a group of lung illnesses characterized by severe inflammation, with no treatment. The fatty acid amide hydrolase (FAAH) enzyme is an integral membrane protein responsible for the hydrolysis of the main endocannabinoids, such as anandamide (AEA). In pre-clinical pain and inflammation models, increasing the endogenous levels of AEA and other bioactive fatty acid amides (FAAs) via genetic deletion or the pharmacological inhibition of FAAH produces many analgesic benefits in several different experimental models. To date, nobody has investigated the role of FAAH inhibition on an ALI mouse model. Mice were subjected to a carrageenan injection and treated orally 1 h after with the FAAH inhibitor URB878 dissolved in a vehicle consisting of 10% PEG-400, 10% Tween-80 and 80% saline at different doses: The inhibition of FAAH activity was able to counteract not only the CAR-induced histological alteration, but also the cascade of related inflammatory events. URB878 clears the way for further studies based on FAAH inhibition in acute lung pathologies.

## 1. Introduction

Acute lung injury (ALI) or its more severe form acute respiratory distress syndrome (ARDS) is a group of lung illnesses characterized by a severe inflammatory process that causes severe hypoxia, hypercapnia, diffuse infiltration, and poor pulmonary compliance [1].

The inflammatory process causes alveolar and interstitial edema, impaired alveolar fluid clearance, reduction in surfactant synthesis and function, and lung fibrosis, depending on whether it starts from the alveolar or microvascular side. The persistence of inflammatory mediators (mainly of neutrophilic origin) in the bronchoalveolar lavage prevents the resolution of the inflammatory process in the lungs [2]. Systemic inflammation is triggered by the release of inflammatory mediators from injured lung tissue, and it can lead to multiple organ failure, which is the leading cause of mortality in ARDS patients. In patients with ALI and ARDS, the intensivist may need to raise their standards to overcome a hypoxia crisis and treat specific pathologies that arise during disease progress. Recruitment movements, prone positioning, nitric oxide inhalation, and extracorporeal membrane oxygenation (ECMO) are all options for resolving a hypoxic crisis [2,3,4,5,6].

Despite significant advances in understanding the biology and pathophysiology of ALI over the past decades, there is still no effective therapy strategy for these disorders [7].

Fatty acid amide hydrolase (FAAH) is an integral membrane protein responsible for the hydrolysis of the endocannabinoid *N*-arachidonoylethanolamide (anandamide, AEA) and other related amidated signaling lipids, such as palmitoylethanolamide (PEA), *N*-oleoylethanolamide (OEA) and linoleoylethanolamide (LEA) [8]. In pre-clinical pain and inflammation models, increasing endogenous levels of AEA and other bioactive fatty acid amides (FAAs) via genetic deletion or the pharmacological inhibition of FAAH produced many analgesic benefits in several different experimental models [8,9,10,11,12,13,14]. 

**Figure 1:**
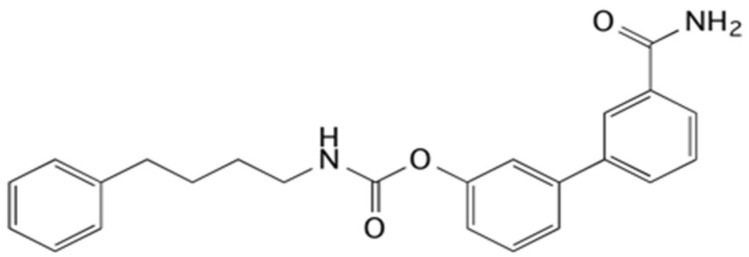
Structural formula of the FAAH inhibitor URB878.

The inhibition of FAAH has been shown to be useful in different experimental models, such as cisplatin- and nicotine-induced vomiting in *Suncus murinus* [15], naloxone-precipitated morphine withdrawal [16], gaping elicited by a lithium-paired context in the rat [17] or chronic cerebral hypoperfusion [18]. Additionally, URB597, the FAAH inhibitor most used as a pharmacological tool [19], has also shown its beneficial effects on other pathologies, such as anxiety and depression [20,21,22,23], neuropathic pain [24], 6-OHDA-induced Parkinson’s model [25], stress [26,27], hypertension [28,29,30,31,32,33,34], and lung problems [35,36,37]. Particular attention is mainly due to the fact that the inhibition of FAAH showed no adverse cardiovascular or gastrointestinal hemorrhaging as is commonly seen with cyclo-oxygenase-2 (COX-2) inhibitors [38,39] and, for this reason, it has become an alternative therapeutic strategy for inflammation-related diseases. In this scenario, the interest in FAAH inhibitors is always very current and worthy of further study. In particular, it was decided to use 4-phenylbutylcarbamic acid 3′-carbamoylbiphenyl-3-yl ester (URB878, Figure 1), an in vitro very potent FAAH inhibitor (IC_50_ = 0.33 ± 0.03 nM) [40] able to increase the knowledge of N-substituted biphenylcarbamic acid FAAH inhibitors [41,42].

URB878 is a structural analog of the pharmacological tool URB597, but differs from it by the N-substituent, which is 4-phenylbutyl in the case of URB878 compared to a cyclohexyl for URB597. The characteristics of the substituents are similar as they are both lipophilic, but the first has an arylalkyl moiety able to improve the interaction with the hydrophobic chain. URB878 has never been studied using in vitro models other than FAAH and in vivo, but the authors chose to use it because they consider it worthy of further study due to its excellent inhibitory capacity. Moreover, it can be considered that the molecule has structural characteristics to be considered selective towards off-targets because they are very similar to those of other FAAH inhibitory carbamates of the URB series, which have shown selectivity, as reported with URB597 and URB937 [19,40,43,44].

The injection of carrageenan (CAR) into the pleural space has been widely used to investigate the pathophysiology of acute inflammation in pleurisy and to assess the role of mediators involved in cellular damage. 

It is a consolidated experimental model that leads to lung injury, local inflammation, infiltration by polymorphonuclear leukocytes (PMNs), mast cell degranulation, the migration and accumulation of pleural exudate, the production of cytokines, oxidative/nitrosative stress and lipid peroxidation with consequences in DNA damage. 

The aim of this study is to investigate, for the first time, the role of FAAH inhibition in acute lung injury. To this end, a new pharmacological tool is used, the sub-nanomolar FAAH inhibitor URB878.

## 2. Results

### 2.1. URB878 Reduces CAR-Induced Lung Injury 

**Figure 2:**
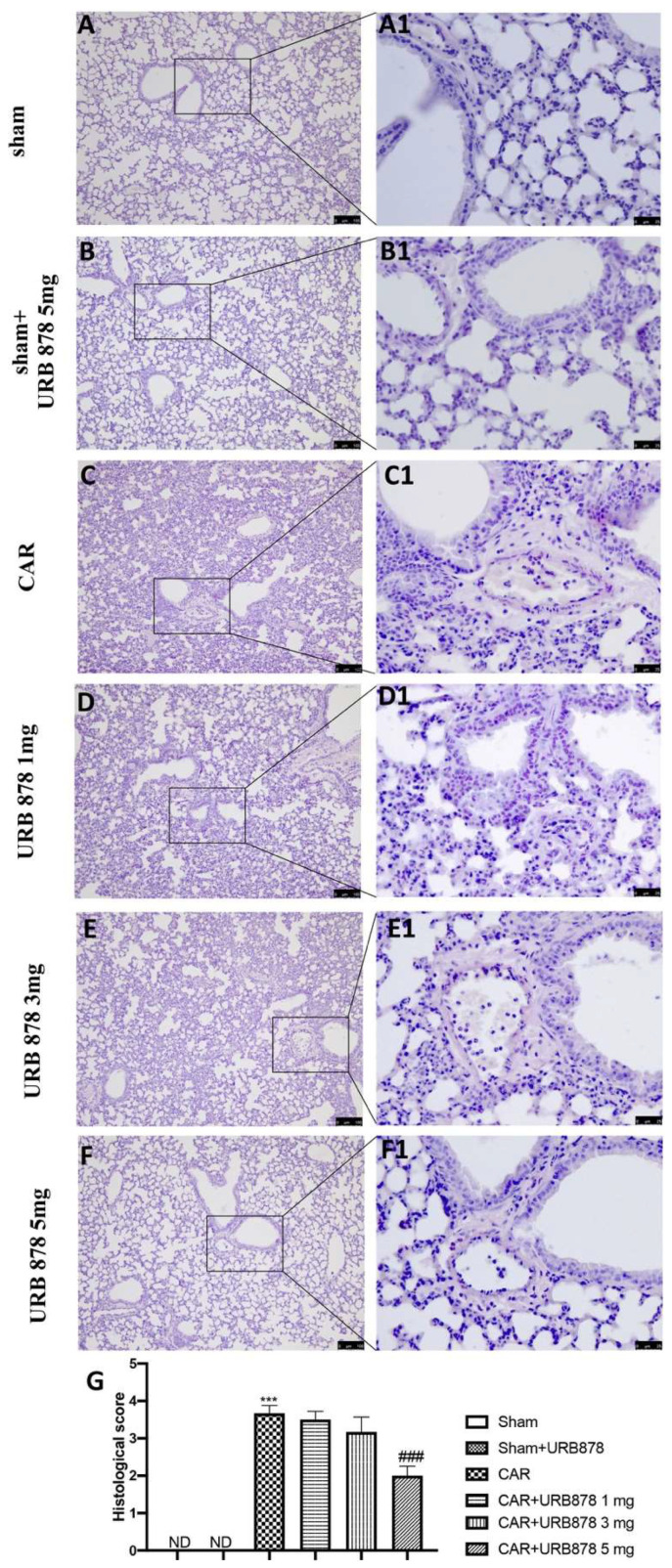
Inhibitions of CAR-induced lung injury by URB878. Sham (**A**,**A1**); Sham+URB878 5 mg/kg (**B**,**B1**); CAR (**C**,**C1**); CAR+URB878 1 mg/kg (**D**,**D1**); CAR+URB878 3 mg/kg (**E**,**E1**); CAR+URB878 5 mg/kg (**F**,**F1**); Histological score (**G**). ND: not detectable. Scale bar for (**A**–**F**) was 100 μm; Scale bar for (**A1**–**F1**) was 25 μm; Values are means ± SEM of 6 mice for all groups. Photos shown are representative of the results obtained. See manuscript for further details. *** *p* < 0.001 vs. sham; ### *p* < 0.001 vs. CAR.

**Figure 3:**
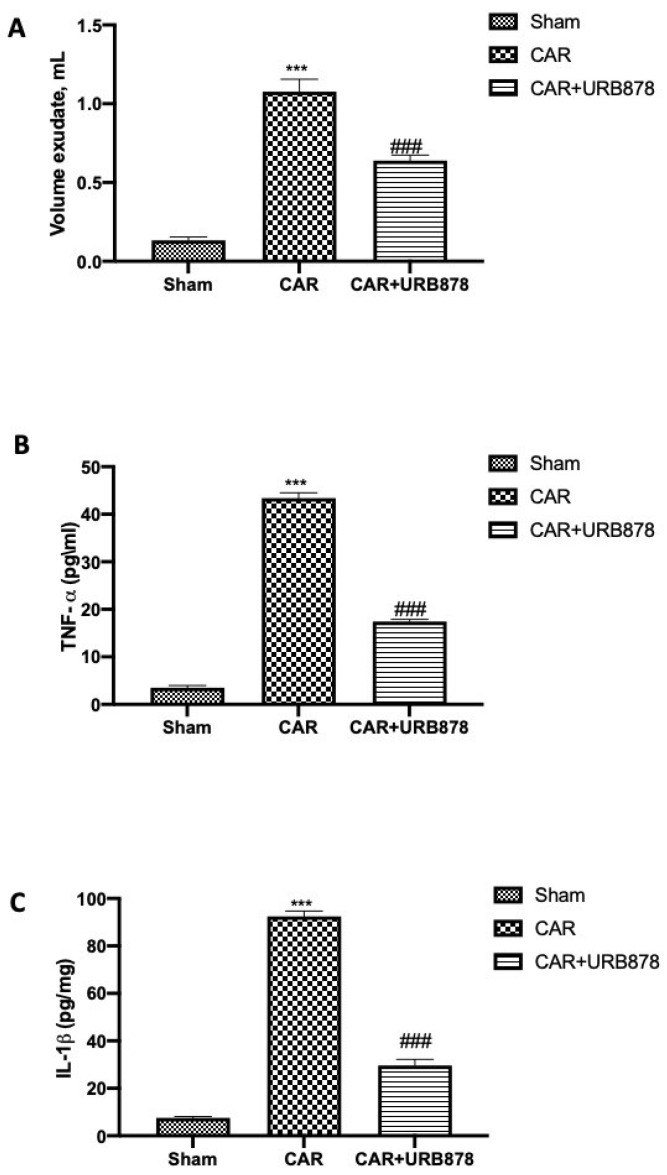
URB878 administration decreases exudate volumes and pro-inflammatory cytokines. Volume exudates (**A**); TNF-α (**B**) and IL-1β (**C**). URB878 was used at a dose of 5 mg/kg. See manuscript for further details. Values are means ± SEM of 6 mice for all groups. *** *p* < 0.001 vs. sham; ### *p* < 0.001 vs. CAR.

The injection of CAR into the pleural cavity was characterized by significant tissue injury and infiltration of neutrophils (PMNs) and edema (Figure 2C,C1; see histological score Figure 2G), compared to sham animals (Figure 2A,A1; see histological score Figure 2G) or to sham animals treated with URB878 at the higher dose (Figure 2B,B1; see histological score Figure 2G). URB878 at the doses of 1 mg/Kg (Figure 2D,D1; see histological score Figure 2G) and 3 mg/Kg (Figure 2E,E1; see histological score Figure 2G) did not show any significant decrease in the inflammatory state. On the other hand, the use of a high dose, that is, 5 mg/kg (Figure 2F,F1; see histological score Figure 2G), showed a significant reduction in the degree of CAR-induced injury. 

Additionally, the injection of CAR stimulated an acute inflammatory response characterized by the accumulation of exudates (Figure 3A) rich in pro-inflammatory cytokine content, such as TNF-α (Figure 3B) and IL-1β (Figure 3C). URB878 at a dose of 5 mg/kg was able to significantly reduce exudate formation as well as pro-inflammatory cytokine production.

### 2.2. URB878 Inhibits CAR-Induced Mast Cell Degranulation, PMN Infiltration and MPO Production 

**Figure 4:**
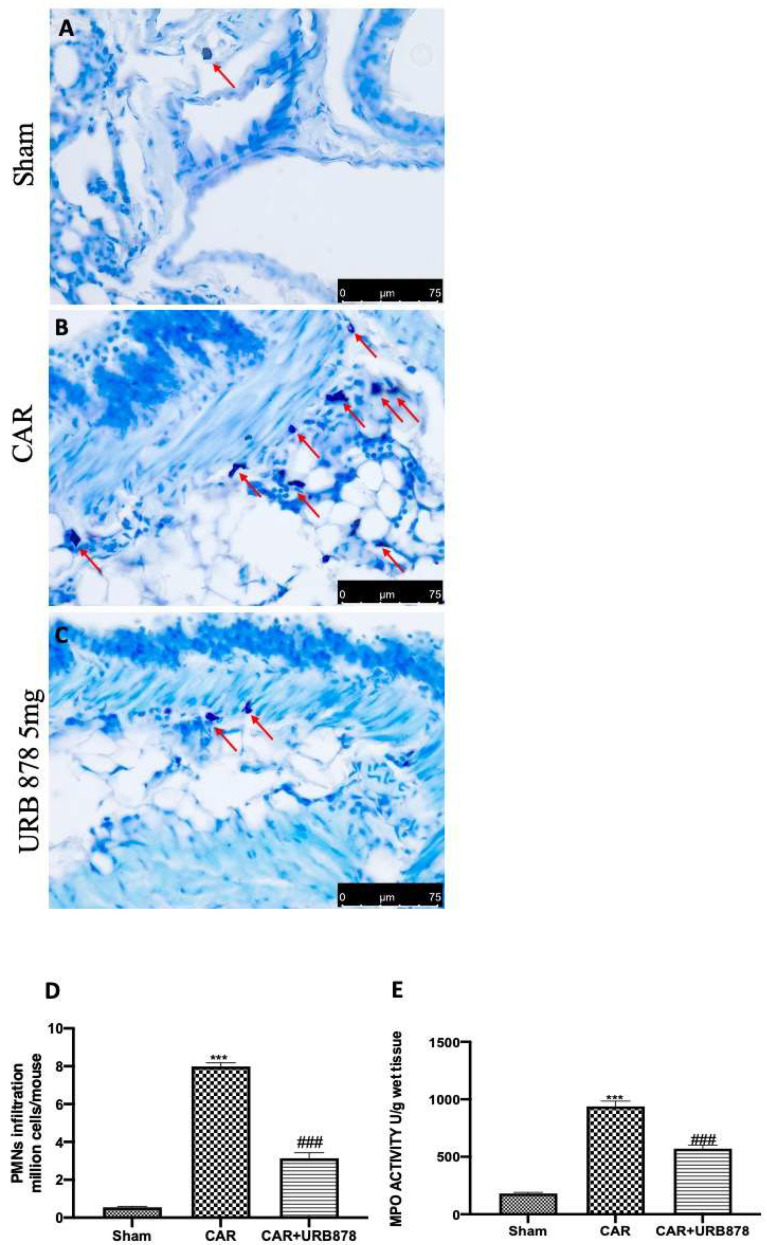
URB878 administration decreases cell infiltration. Sham (**A**); CAR (**B**); CAR+URB878 5 mg/kg (**C**); PMN infiltration (**D**); MPO levels (**E**). Mast cells were indicated with red arrow. Scale bar for (**A**–**C**) was 75 μm; Values are means ± SEM of 6 mice for all groups. Values are means ± SEM of 6 mice for all groups. Photos shown are representative of the results obtained. See manuscript for further details. *** *p* < 0.001 vs. sham; ### *p* < 0.001 vs. CAR.

Lung edema, epithelial and endothelial injury are strongly connected with a significant influx of mast cells, PMNs, and leukocytes into the interstitium and broncheoalveolar space, which alters lung health [45,46,47]. After the CAR injection, we found a significantly increase in mast cell degranulation, PMN infiltration and MPO levels (Figure 4B,D,E), compared to sham animals (Figure 4A,D,E). On the other hand, the inhibition of FAAH with 5 mg/kg of URB878 significantly decreased all these parameters (Figure 4C–E). 

### 2.3. URB878 Reduces the Expressions of the Adhesion Molecules ICAM and P-Selectin 

**Figure 5:**
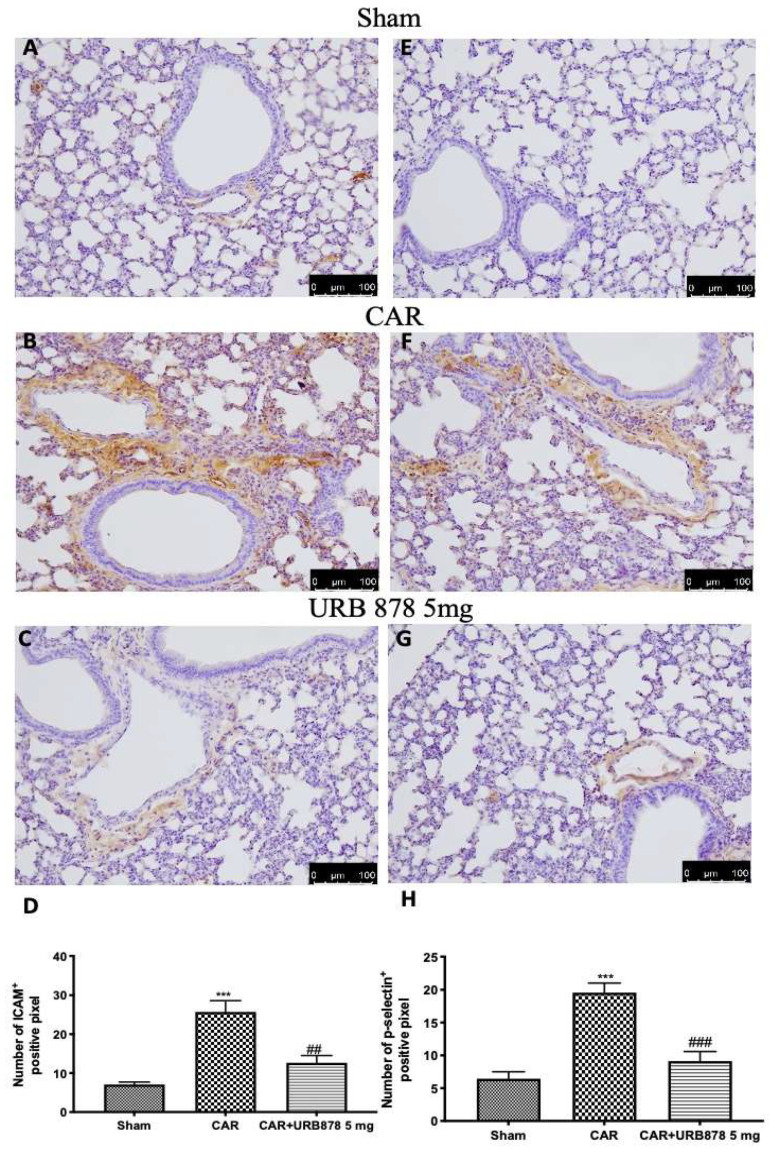
URB878 administration decreases ICAM and P-selectin expression. Sham (**A**); CAR (**B**); CAR+URB878 5 mg/kg (**C**); graphical analysis (**D**) for ICAM. Sham (**E**); CAR (**F**); CAR+URB878 5 mg/kg (**G**); graphical analysis (**H**) for P-selectin. Scale bar for (**A**–**C**) and (**E**–**G**) was 100 μm; Values are means ± SEM of 6 mice for all groups. Photos shown are representative of the results obtained. See manuscript for further details. *** *p* < 0.001 vs. sham; ## *p* < 0.01 vs. CAR; ### *p* < 0.001 vs. CAR.

The intensity of ICAM and P-selectin staining significantly increases in lung tissue slices taken from CAR-treated animals (Figure 5B,F; see Figure 5D and Figure 5H, respectively) compared to sham groups (Figure 5A,E; see Figure 5D and Figure 5H, respectively). URB878 at a dose of 5 mg/kg significantly decreases both stainings (Figure 5C,G; see Figure 5D and Figure 5H, respectively).

### 2.4. Effect of URB878 Administration on CAR-Induced Nitrosative Stress 

**Figure 6:**
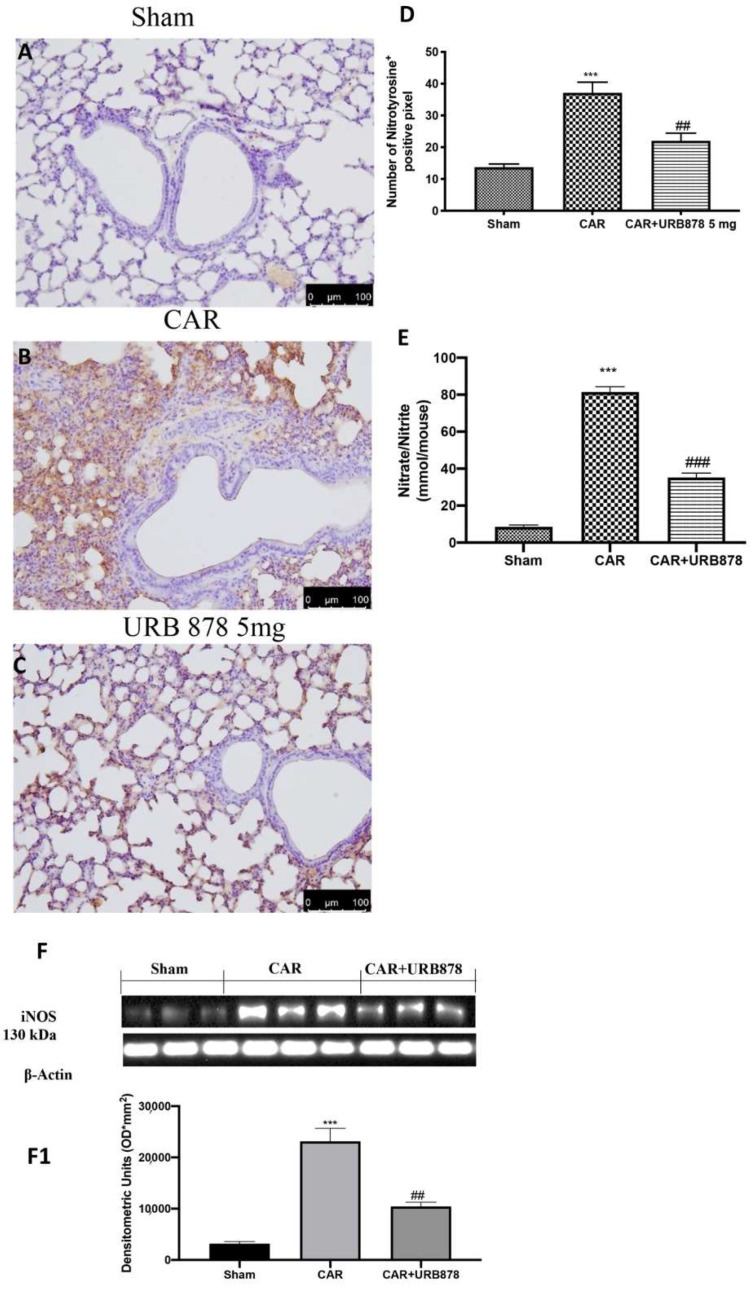
Effect of URB878 on nitrosative stress. Sham (**A**); CAR (**B**); CAR+URB878 5 mg/kg (**C**); graphical analysis (**D**) for nitrotyrosine. Nitrite/nitrate levels (**E**); Western Blot for iNOS expression (**F**); densitometric analysis (**F1**); Scale bar for (**A**–**C**) was 100 μm; Values are means ± SEM of 6 mice for all groups. Photos shown are representative of the results obtained. See manuscript for further details. *** *p* < 0.001 vs. sham; ## *p* < 0.01 vs. CAR; ### *p* < 0.001 vs. CAR.

By Western blots, immunohistochemistry and nitrite/nitrate analyses, we investigated the effects of FAAH inhibition on nitrosative stress. We found that, after the CAR injection, there was a significant increase in nitrotyrosine (Figure 6B,D), iNOS (Figure 6F; see densitometric analysis Figure 6F1) and nitrite/nitrate levels (Figure 6E), compared to the sham group (Figure 6A; see Figure 6D for nitrotyrosine expression; Figure 6F for densitometric analysis; Figure 6F1 for iNOS activity; Figure 6E for nitrite/nitrate levels). As assumed, the inhibition of FAAH by URB878 protects the lung against nitrosative stress, decreasing free radical production (Figure 6C; see Figure 6D for nitrotyrosine expression; Figure 6F for densitometric analysis; Figure 6F1 for iNOS activity; Figure 6E for nitrite/nitrate levels). 

### 2.5. FAAH Inihibition Reduces DNA Damage and Lipid Peroxidation

**Figure 7:**
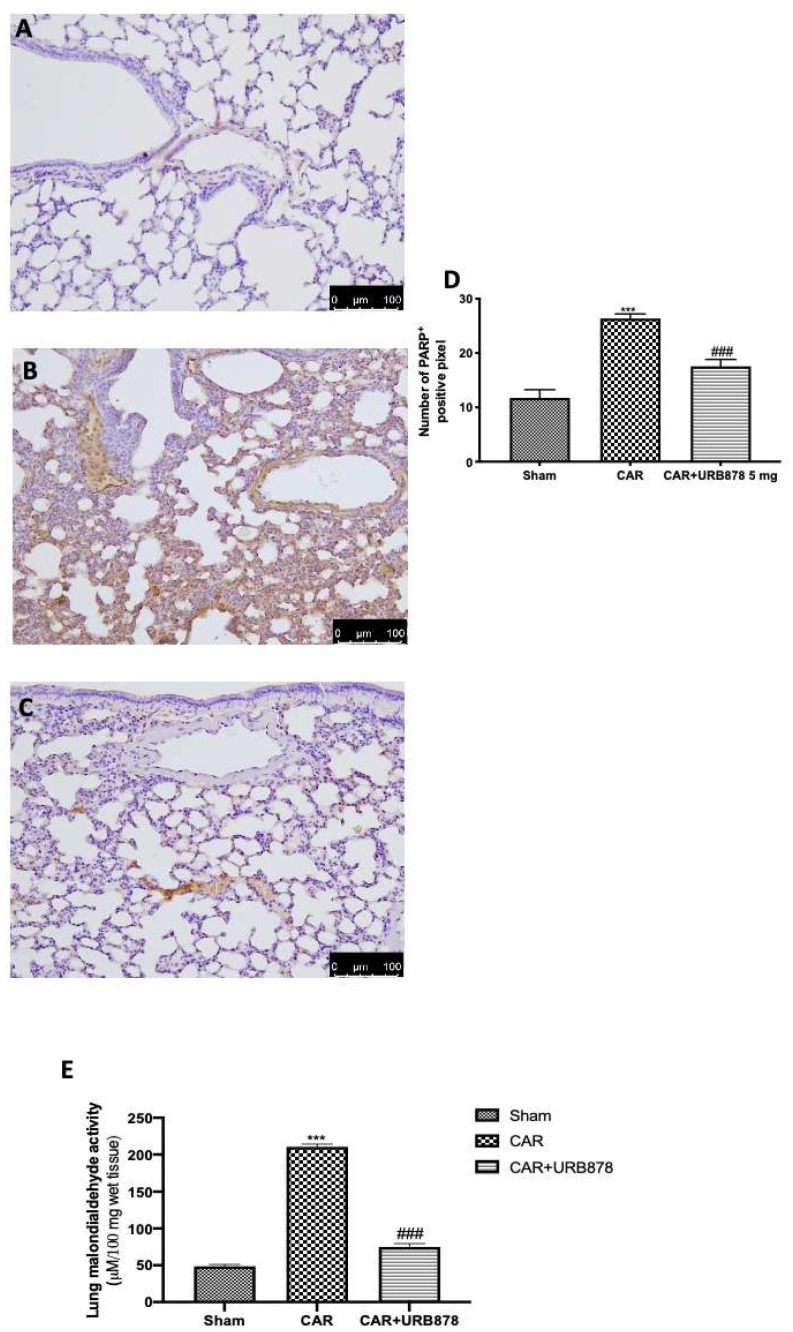
Effect of URB878 on PARP expression and MDA levels. Sham (**A**); CAR (**B**); CAR+URB878 5 mg/kg (**C**); graphical analysis (**D**) for PARP. Malondialdehyde activity levels (**E**); Scale bar for (**A**–**C**) was 100 μm; Values are means ± SEM of 6 mice for all groups. Photos shown are representative of the results obtained. See manuscript for further details. *** *p* < 0.001 vs. sham; ### *p* < 0.001 vs. CAR.

Immunohistochemical analysis of lung tissue collected from CAR-treated mice showed an increased in PARP positive cells (Figure 7B,D) compared to the sham group (Figure 7A,D). URB878 at a dose of 5 mg/kg was able to significantly decrease PARP expression (Figure 7C,D). Additionally, we evaluated lipid peroxidation by MDA activity, and we found that CAR injection in the pleural cavity significantly increased lipid peroxidation compared to the sham group (Figure 7E). On the other hand, URB878 at a dose of 5 mg/kg significantly decreased lipid peroxidation levels.

### 2.6. URB878 Reduces CAR-Induced Lung Inflammation 

**Figure 8:**
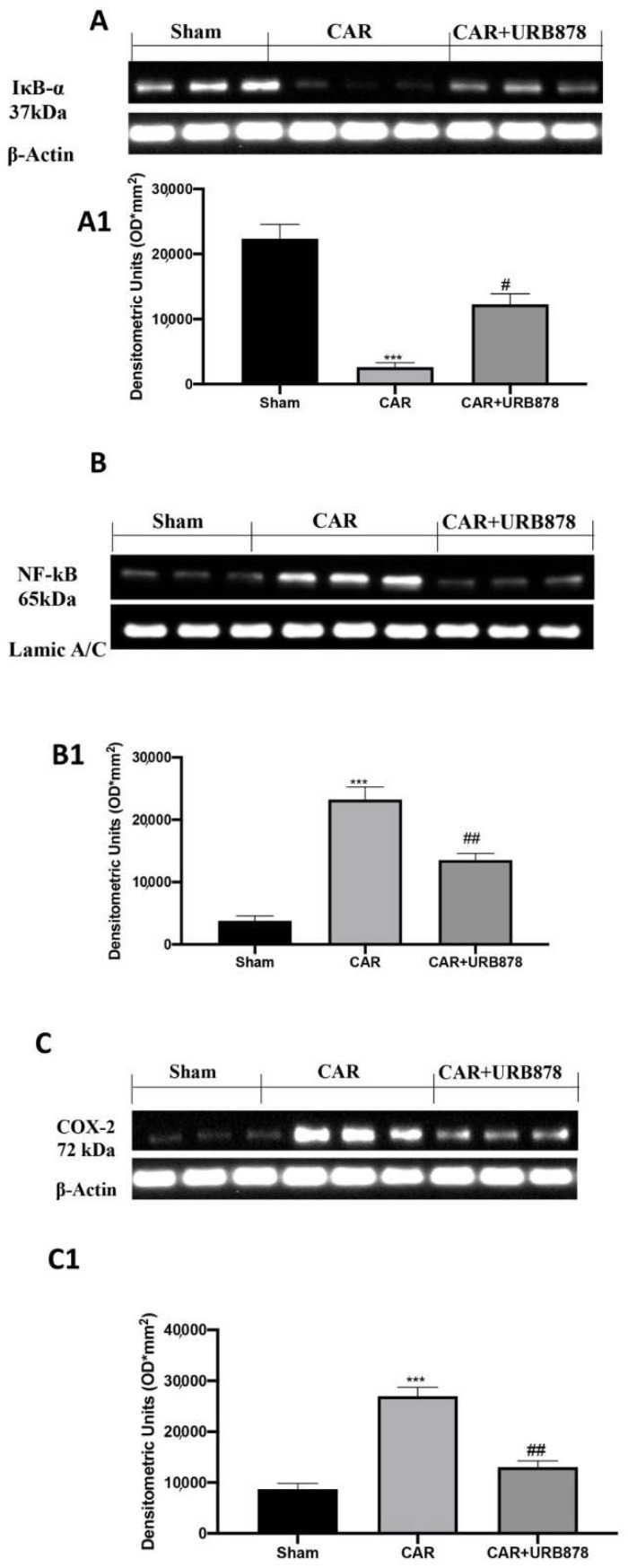
Western Blot and relative densitometric analysis for IkB-α (**A**,**A1**) NF-kB (**B**,**B1**) and Cox-2 (**C**,**C1**). Values are means ± SEM of 6 mice for all groups. See manuscript for further details. *** *p* < 0.001 vs. sham; # *p* < 0.05 vs. CAR; ## *p* < 0.01 vs. CAR.

At the end of the experiment, which lasted 4 h, the lungs were processed using Western blots. The basal expression of IkB-α was significantly decreased in the lung samples obtained from CAR-injected mice, compared to the sham group (Figure 8A; see densitometric analysis in Figure 8A1). URB878 at a dose of 5 mg/kg prevented CAR-induced IkB-α degradation. Moreover, CAR increased NF-kB levels in the nuclear fractions compared with the sham-treated mice URB878 at a dose of 5 mg/kg, whose administration significantly reduced the levels of NF-kB (Figure 8B; see densitometric analysis in Figure 8B1). Furthermore, we investigated the levels of COX-2 expression following URB878 treatment and found that, after CAR injection, there was a significantly increase in lung tissue. After the treatment, COX-2 expression was reported have reached physiological levels (Figure 8C; see densitometric analysis in Figure 8C1). 

## 3. Discussion and Conclusions

FAAH is a member of a broad serine hydrolase family that hydrolyze NAEs, such as AEA, PEA, and OEA, and this action is known as an entourage effect. The increased levels of NAEs due to the genetic or pharmacological inhibition of FAAH have been related to have extensive beneficial effects in both acute and chronic situations [48,49,50,51,52,53,54,55,56]. As a result, further research into FAAH inhibitors is needed, as well as the investigation of molecular pathways involved in the inflammatory response. 

The hyperactivation of the immune system is typically linked to ALI, resulting in pulmonary inflammation, pulmonary edema, alveolar destruction, and, in severe cases, respiratory failure [57,58]. There is a significant rate of death linked with ALI/ARDS because no pharmaceutical medications have been licensed by the FDA to treat it. Animal models of ALI have greatly aided our understanding of the pathogenesis and pathophysiology of the ALI and ARDS clinical syndromes. One of the most used models to reproduce ALI damage is pleurisy obtained with an injection of carrageenan into the pleural space [59,60,61]. To date, despite the numerous studies carried out with FAAH inhibitors or with the use of genetically modified animals, no one has evaluated the role of FAAH in an experimental model of carrageenan-induced ALI. 

The infiltration of inflammatory cells and rupture of the alveolar–capillary barrier histologically characterize the acute phase of ALI, resulting in a proteinaceous exudate that floods the alveolar passages, impairing gas exchange and precipitating respiratory failure [62,63]. In our study, we found that, with the inhibition of the FAAH enzyme, the entire histological damage decreases in a dose-dependent manner. The production of proinflammatory cytokines, such as TNF-α and IL-1β, is required for lung neutrophil recruitment and subsequent lung damage. TNF-α and IL-1β are two of the first cytokines generated by alveolar macrophages during the inflammatory phase of lung damage [64,65]. These cytokines, as well as other proinflammatory compounds, initiate, amplify, and perpetuate the inflammatory response during acute lung injury [66,67,68,69,70]. The inhibition of FAAH enzyme by URB878 at the dose of 5 mg/kg significantly decreases both cytokines release as well as the volume of exudates. Transepithelial cellular migration is an important feature of ALI, as well as the degranulation of mast cells [71,71,72]. Our study demonstrates, for the first time, that the stabilization of MCs and neutrophils from degranulation and migration by the inhibition of FAAH could attenuate inflammatory response. Inflammatory processes are aided by P-selectin and intercellular adhesion molecule-1 (ICAM-1). ALI is related, through the increase in leukocyte adherence, to the endothelium of the vascular wall [73]. FAAH inhibition significantly reduces the expression of both molecules. Different isoforms of nitric oxide synthases (NOS) and nitrosative stress determinants play essential roles in the pathogenesis of ALI-induced pulmonary dysfunction [74]. In our study, and according to the literature, we found a significant increase in nitrite and nitrate levels as well as in iNOS and nitrotyrosine expression. FAAH inhibition attenuated this increase, bringing expressions back to physiological levels. In addition, the injection of CAR into the pleural cavity triggers other cytotoxic effects, including DNA damage as well as lipid peroxidation [75]. In our study, we found that FAAH inhibition by URB878 at the dose of 5 mg/kg was able to reduce PARP activation as well as lipid peroxidation. Pulmonary expression of these mediators, especially TNF-α and IL-1β, has been linked to the nuclear translocation of the transcription factor NF-κB [76,77]. A lot of stimuli can cause IκBα phosphorylation, with the consequent nuclear translocation of the NF-κB subunit p65, which leads to a transcription of multiple proinflammatory genes, chemokines, and adhesion molecules [78,79]. In our study, we found a significant increase in NF-κB activation as well as in COX-2 compared to the sham group. On the other hand, FAAH inhibition significantly decrease the activation on this pathway.

In conclusion, our findings confirm the protective effect of FAAH in the development of CAR-induced pleurisy by inhibiting NF-κB activation, as well as the expression of proinflammatory cytokines. These actions could explain why neutrophil migration, as well as nitrosative stress, decreases during inflammatory response. The findings of this study, taken combined, add to our understanding of the FAAH modulation in the pathophysiology of inflammation and support its therapeutic potential for lung inflammatory diseases.

## 4. Materials and Methods

### 4.1. Animals

CD1 mice (25–30 g, Envigo, Milan, Italy) were employed. The University of Messina Review Board for animal care (OPBA) approved the study (Prot. Num. 266/2021-PR). All animal experiments agree with the new Italian regulations (D.Lgs 2014/26), E.U. regulations (E.U. Directive 2010/63) and the ARRIVE guidelines. ALI was made as previously described by Di Paola et al. [80].

### 4.2. Experimental Design and Groups

Briefly, mice were anaesthetized and saline (0.1 mL) or saline containing 2% λ carrageenan (0.1 mL) was injected into the pleural cavity. 

Mice were casually distributed into the following groups:

(1) CAR: mice were subjected to the CAR injection described above, and treated with saline solution;
(2) CAR+URB878: mice were subjected to the CAR injection described above, and treated orally 1 h after with URB878 dissolved in a vehicle consisting of 10% PEG-400, 10% Tween-80 and 80% saline at different doses;
(3) Sham groups: animals were subjected to an injection of saline solution;
(4) Sham groups+URB878: animals were subjected to URB878 treatment at different doses (data not shown) (in our results, there were not significant difference observed between Sham and Sham+URB878).

After four hours, the animals were killed, and the lungs were conserved for further study. Doses were chosen based on a dose–response study carried out in our lab. URB878 at the doses of 1 mg/kg and 3 mg/kg did not show any advantage in CAR-induced lung inflammation. For this reason, we conducted our experiment only with the dose of 5 mg/kg.

### 4.3. Exudate and Leukocytes Count

The measure of exudates and count of leukocytes was performed as previously described [81]. Briefly, at the end of the experiment, the chest was opened, and the pleural cavity washed with 1 mL of saline solution containing heparin and indomethacin. The exudate and washing solution were removed by aspiration and the total volume measured. 

The leukocytes in the exudate were suspended in phosphate-buffer saline (PBS) and, after blue toluidine staining, counted with an optical microscope in a Burker’s chamber.

### 4.4. Western Blot Analysis of Cytosolic and Nuclear Extracts

Extracts of the cytosol and nucleus were prepared, as previously mentioned [82,83,84,85,86]. The following primary antibodies were used: anti-iNOS (1:500, Santa Cruz Biotechnology, #sc-7271, Dallas, TX, USA), anti-Cox-2 (1:500, Santa Cruz Biotechnology, #sc-19999), anti-FAAH (1:500, Sigma-Aldrich Corp., Milan, Italy), anti-Iκbα (1:500, Santa Cruz Biotechnology, #sc-1643), and anti-nfκb (1:500, Santa Cruz Biotechnology, #sc8414) in 1 × PBS, 5% *w*/*v* non-fat dried milk, and 0.1% Tween 20, at 4 °C overnight [87,88,89,90]. For the cytosolic fraction, Western blots were also explored with antibody against β-actin protein (1:500, Santa Cruz Biotechnology, Dallas, TX, USA). The same methods were used for nuclear fraction with lamin A/C (1:500, Sigma-Aldrich Corp., Milan, Italy) [91,92]. Signals were examined with an enhanced chemiluminescence (ECL) detection system reagent, according to the manufacturer’s instructions (Thermo, Monza, Italy). The relative expression of the protein bands was quantified by densitometry with BIORAD ChemiDoc^TM^ XRS+ software (Hercules, CA, USA) [83,87,93,94,95].

### 4.5. Evaluation of Tissue Lipid Peroxidation

Lipid peroxidation were assessed with the consolidated method of malonaldehyde, as previously described, and expressed as μM MDA/mg of proteins [96].

### 4.6. Cytokine and Nitrite/Nitrate Measurement

TNF-α, IL-1β and nitrite/nitrate concentration were measured in the exudates using ELISA kits (R & D Systems, Minneapolis, MN, USA), following the manufacturer’s instructions, and expressed as pg/mL, or with a Griess reaction assay kit and expressed as mmol/mouse [80,93,94,96,97,98,99,100,101].

### 4.7. Histopathological Evaluation with Hematoxylin/Eosin and Toluidine Blue

The lung tissues were stained with hematoxylin and eosin (H & E) for architecture alterations and toluidine blue for mast cell degranulation, and analyzed using a light microscopy (Leica DM6, Milan, Italy) associated with an Imaging system (LasX Navigator, Milan, Italy). The degree of inflammation was evaluated according to a score from normal (0) to severe (4), as previously described [83,87,102]. 

### 4.8. Immunohistochemical Localization of Nitrotyrosine, Poly(ADP-ribose), ICAM and P-Selectin

At the end of the experiments, lung tissues were incubated with anti-ICAM-1 murine polyclonal antibody (1/100 in PBS, *v*/*v*, Santa Cruz Biotechnology), anti-P-selectin murine polyclonal antibody (1/100 in PBS, *v*/*v*, Santa Cruz Biotechnology), anti-PAR murine polyclonal antibody (1/100 in PBS, *v*/*v*, Santa Cruz Biotechnology) and anti-nitrotyrosine rabbit polyclonal antibody (1:200 in PBS, *v*/*v*, Millipore), as previously described [72,88,103,104,105,106,107,108]. Immunohistochemical images were collected using (Leica DM6, Milan, Italy) associated with an Imaging system (LasX Navigator, Milan, Italy). The digital images were opened in ImageJ, followed by deconvolution using the color deconvolution plug-in. When the IHC profiler plug-in is selected, it automatically plots a histogram profile of the deconvoluted DAB image, and a corresponding scoring log is displayed. The histogram profile corresponds to the positive pixel intensity value obtained from the computer program. All immunohistochemical analyses were carried out by two observers blinded to the treatment [72,88,103,104,105,106,107,108,109].

### 4.9. Materials

Unless otherwise stated, all compounds were purchased from Sigma-Aldrich.

### 4.10. Statistical Evaluation

In this study, the data are expressed as the average ± SEM and represent at least 3 experiments carried out in different days. For in vivo studies, N represents the number of animals used. The number of animals used for in vivo studies was carried out by G*Power 3.1 software (Die Heinrich-Heine-Universität Düsseldorf, Düsseldorf, Germany). Data were analyzed by an experienced histopathologist, and all the studies were performed without knowledge of the treatments. The results were analyzed by one-way ANOVA followed by a Bonferroni post hoc test for multiple comparisons. A *p* value of less than 0.05 was considered significant.

### 4.11. Synthesis of URB878

The FAAH inhibitor URB878 was synthesized as previously reported [40]. Briefly, to a stirred solution of 3′-carbamoylbiphenyl-3-ol in toluene, 4-phenylbutylisocyanate was added (twice) and the reaction was refluxed for 8 h, then cooled and concentrated. The residue was purified by column chromatography and recrystallization from ethanol was performed to obtain the desired final compound.

## Data Availability

The data used to support the findings of this study are available from the corresponding author upon request.
